# Sulfate Deficiency as a Risk Factor for Autism

**DOI:** 10.1007/s10803-019-04240-5

**Published:** 2019-09-27

**Authors:** Richard J. Williams

**Affiliations:** Rybett Controls, Inc, 21615 Tribune St., Chatsworth, CA 91311 USA

**Keywords:** Autism, Sulfate, Pregnancy, Drinking water, Beverages

## Abstract

This is a study of water and beverages consumed during pregnancy by mothers of children with autism. Materials included vials for water samples and a survey to describe the water and beverages. Samples were tested for sulfate and surveys evaluated for average daily levels. Results were stratified for selected regions of the United States. Areas with the highest rates of autism showed a trend toward lower levels of sulfate compared to areas with low rates of autism (28% sulfate, n = 45, p = 0.059). Severe autism was associated with low sulfate levels while mild symptoms were associated with higher levels of sulfate (− 0.32 correlation, n = 86, p < 0.01). The results suggest that sulfate may be helpful in reducing both the incidence and severity of autism.

**Electronic supplementary material:**

The online version of this article (10.1007/s10803-019-04240-5) contains supplementary material, which is available to authorized users.

## Introduction

Autism spectrum disorders (ASD) affect social interaction, communication, behavior and the senses. In the United States, the prevalence is 1 in 59 for all children and 1 in 37 for boys based on data from the Centers for Disease Control and Prevention (Baio et al. 2014). The cause is not well understood although genetic predisposition coupled with environmental influence is strongly suspected (Lyall et al. 2017). It is the purpose of this study to evaluate sulfate levels in drinking water and beverages as a risk factor for autism. It is our goal to better understand the causes of autism and illuminate possible preventative measures.

One characteristic of autism is dysfunctional sulfur metabolism. In particular, the oxides of sulfur are implicated: sulfur dioxide, sulfite and sulfate $\left( {{\text{SO}}_{2} ,\;{\text{SO}}_{3}^{2 - } \;{\text{and}}\;{\text{SO}}_{4}^{2 - } } \right)$. Sulfate may be ingested directly or it may be produced as an end product of the transsulfuration pathway. In this pathway, the amino acid methionine contributes sulfur dioxide and sulfite which is finally oxidized by sulfite oxidase enzyme to become sulfate. An English study reports the urine of those with autism contains 50 times the sulfite and double the sulfate of neurotypicals (Waring and Klovrza 2000). In an Arizona study that investigated blood sulfate levels in a cohort with autism, free sulfate was 35% and total sulfate was 72% of non-autistic individuals (Adams et al. 2011). In addition, a French study of nasal stem cells found decreased expression of either the molybdenum cofactor sulfurase or aldehyde oxidase genes (MOCOS or AOX) in 91% of a small group (n = 11/12) of autistic participants (Feron et al. 2016). Both of these genes are part of the molybdenum cofactor pathway, responsible for sulfite oxidase enzyme, among several others. Impaired sulfite oxidase production results in an increase of sulfite as noted above.

Sulfate is a common nutrient and functions in a variety of chemical processes including the development of tissue structure for important organs. During human pregnancy, maternal circulating sulfate levels double during the final trimester. This highlights the importance of sulfate in fetal development (Dawson et al. 2015). In particular, heparan sulfate is essential for neuron regulation. In studies of mice with compromised heparan sulfate synthesis, symptoms similar to those found in autism resulted, including impairments in social interaction, expression of repetitive behavior and difficulties with vocalization (Irie et al. 2012). In humans, the examination of postmortem brain tissue in young individuals showed reduced levels of heparan sulfate for those with autism vs neurotypicals (Pearson et al. 2013). Finally, sulfate supports sulfonation and sulfotransferase enzymes which help to remove xenobiotics and certain pharmacological drugs. Through a sulfonate intermediary, 3′-phosphoadenosine 5′-phosphosulfate (PAPS), sulfate is attached to unwanted chemicals increasing water solubility to facilitate removal (Gamage et al. 2005). Without sufficient sulfate, developing children may be at heightened risk from xenobiotics and environmental factors that require metabolism via sulfonation.

With the importance of sulfate in mind, the prevalence of autism was researched using Department of Education data as required by IDEA legislation, the Individuals with Disabilities Education Act (US DoE 2010). For children aged 6 through 11 in 2010, prevalence was calculated for all 50 states. The states with the highest rates of autism were Minnesota, Maine, Oregon and Connecticut. States with very low rates included Iowa, Colorado, Oklahoma, Montana, Kansas and South Dakota. Water Quality Reports for the major cities in each of these states were accessed for data on sulfate levels. Sulfate reporting is not required by the federal government and not all cities include this information in their annual water reports. For the ten states mentioned above, sufficient data was available and sulfate levels were averaged with population as a weighting factor. The four states with a high incidence of autism averaged 13 mg/L sulfate. The six states with low incidence averaged 113 mg/L. This difference prompted our research study (Williams 2017).

## Methods

The focus of our investigation was drinking water and beverages that were consumed during pregnancy by mothers of children with autism. Toward this end, water kits were created containing two sample vials, a survey sheet, a consent form, a background explanation and a postage prepaid return box. Participants were recruited via Facebook by boosting an invitation post to a targeted audience, adult women with an interest in autism. Water kits were mailed to those with children on the autism spectrum willing to provide a shipping address. Participants were instructed to sample the two most important water sources during their pregnancy. Practically speaking, this required that they currently live in the same place where they gave birth. On the survey, participants identified their sampled water, bottled water and beverages like coffee, tea, milk, wine, beer, juice and soda. They estimated the daily number of 8 oz glasses for each liquid. Finally, they provided an estimate of the severity of their child’s condition.

### Participants

In addition to the screen described above (adult females with an interest in autism), three specific regions within the United States were targeted. These regions were the 8 states with the highest prevalence of autism, the eight states with the lowest prevalence and the southwest region including Southern California and Central Arizona. As indicated earlier, the high prevalence states seemed to have low levels of sulfate in public water while lower prevalence states had much higher. We wanted to confirm this disparity and learn what we could from these two poles of autism. The southwest was chosen because it is served by Colorado River water which is high in minerals and sulfate. If autism is indeed correlated with low levels of sulfate, then mothers of children with autism in this region must have avoided the high sulfate water. If not, the premise of the study is proven false. Statistics for participants in these regions plus the full USA are summarized in Table 1. Prevalence weighted by population was calculated from Department of Education data as before (US DoE 2010).

**Table 1:** Details of participants by region

Participants by region (Mothers of children with autism)				
Region	Description	n	Boys (%)	Mean age (range)
High prevalence (1 in 84)	Minnesota, Maine, Oregon, Connecticut, Pennsylvania, Massachusetts, New Jersey, Indiana	22	73	6.5 years (2–16 years)
Low prevalence (1 in 234)	Iowa, Louisiana, Colorado, Oklahoma, New Mexico, Montana, Mississippi, Kansas	23	65	6.6 years (2–16 years)
Southwest region	Southern California and Central Arizona served by Colorado River water	32	81	5.4 years (2–18 years)
Other	Other states outside of the target regions	9	100	4.9 years (2–9 years)
Total	All of the United States combined from above	86	77	5.9 years (2–18 years)

### Statistical Analyses

An important consideration is bias in the target population. Participants were recruited in English via Facebook and required to read and complete two sheets of paper: an Informed Consent Form and a Survey Form consisting of instructions and 24 spaces for data entry. At first glance, it might have appeared to be a formidable task, especially since the forms concerned behavior many years past. For this reason, it seems likely that the resulting study population was above average in intelligence or education, in addition to being literate in English. It is difficult to judge whether or not this produced a significant bias regarding drinking water or beverage choices. However, since sulfate content is not well publicized, we suspect any bias was minimal.

All collected data was stored in an Open Office spreadsheet version 4.1.5 by Apache Software. Statistical calculations were performed by the functions AVERAGE, MEDIAN, STDEV, VAR, TTEST, CORREL and FORECAST. Linear regression lines, Pearson correlation coefficients and null hypothesis probabilities are presented in the results section. Graphs were generated within the word processor with manually entered values.

### Sulfate Measurements

Water samples were analyzed using a Hanna Instruments Model HI 96751C digital photometer. The resolution is 1 mg/L with an accuracy of ± 5% of the reading. The range is 0 to 150 mg/L with higher samples diluted with distilled water to bring them back into range. In operation, the meter is zeroed with a 10 mL water sample in a glass cuvette. Then barium chloride is added to cause a barium sulfate precipitate which clouds the solution. The drop in light transmission is measured and then used by an onboard microprocessor to calculate the concentration of sulfate in the water. This method is based on the turbidometric assay previously described by Jackson and McCandless in the journal of Analytical Biochemistry (Jackson and McCandless 1978).

### Supporting Data

After the water samples were tested, the survey form was evaluated to figure the average sulfate concentration of the water actually consumed by the participant. A typical mother drinks a few glasses of tap or well water, which may be unfiltered or filtered. Reverse osmosis filters and multi-stage units like Zero Water remove nearly all sulfate. Simple carbon filters such as Brita pitchers or those in refrigerators reduce chlorine but leave sulfate virtually untouched. Tests using the Hanna photometer showed Brita and GE refrigerator carbon filters removed between 6 and 9% of sulfate. Zero Water performed as advertised and reduced sulfate to < 1 mg/L. Tap water is usually supplemented with bottled or flavored water of various local and national brands. The sulfate levels of bottled waters were determined by a variety of means including published water quality reports (no statistics given) or sample testing using the Hanna photometer (single samples only). Common bottled waters are listed in Table 2. For clarity, bottled water is divided into two classes separated by the Environmental Protection Agency (EPA) published median for sulfate in public tap water, which is 24 mg/L (US EPA 2003).

**Table 2:** Sulfate concentration of common bottled water and beverages

Sulfate in bottled water and beverages (From quality reports, photometer tests and Florin’s paper)	
Liquid class	Brands (mg/L sulfate)
Bottled water, low (sulfate < 24 mg/L) Single reported value	Aquafina (0), Crystal Geyser (1), Dasani (10), Fiji (2), La Croix (0), Nestle Pure Life (16), Niagara (15), Ozarka (3), Poland Spring (5), Safeway (0), Sam’s Choice (0), Smart Water (0), Sparkletts (3), Vitamin Water (1), Volvic (7)
Bottled water, high (sulfate > 24 mg/L) Single reported value	Arrowhead (42), Calistoga (110), Contrex (430), Gerolsteiner (38), Manitou Mineral (120), Pellegrino (408), Penafiel (130), Perrier (46), Pure Montana (148), Starkey (140), Vittel (306)
Beverages per Florin (mean values from four or more samples)	Beer, lager (47), Coffee, purified water (100), Juice, apple or citrus (70), Juice, grape (200), Milk, cow or soy (100), Soda, cola (80), Soda, non-cola (40), Tea (100), Wine (300)

The table also includes sulfate estimates for beverages from an Australian study (Florin et al. 1993). The table values present averages from four or more samples but have wide variations since they cover beverages from a range of products. The concentrations shown for coffee and tea assume they are brewed with purified water. This is accurate for major coffee shops such as Starbucks and McDonalds as they advertise the use of highly purified water. For home brews, the sulfate content of the water must be added to that shown for coffee and tea flavoring.

## Results

Participants were recruited for the study beginning in May of 2018 and the final water kit was evaluated in October. The best response occurred over the summer when school was out. The results are summarized by *Region* in Table 3. To recap, the Southwest region included Central Arizona and Southern California, both served by a combination of Colorado River water and local sources. For this region, sulfate levels are much higher than the national norm since Colorado River water is typically in the 250 mg/L range. The Lowest and Highest Prevalence regions each consisted of the eight states with the lowest and highest rates of autism per Department of Education data. Finally, all of the regions were merged with participants from the rest of the country for a look at the USA as a whole.

**Table 3:** Sulfate data collected from mothers in drinking water study

Sulfate in drinking water and beverages (Mothers of children with autism during pregnancy)							
Region	Autism severity	n	Tap water (mg/L)	Water mix (mg/L)	SD (mg/L)	Bev & water (mg)	SD (mg)
Southwest (AZ and CA)	Mild	14	136	53.9	44.6	234	133
	Mod	13	183	37.3	27.3	185	138
	Severe	5	114	6.5	7.9	97	70
	All	32	151	39.8	37.5	193	133
Lowest prevalence	Mild	8	199	82.3	98.5	242	150
	Mod	13	43	26.6	23.7	138	94
	Severe	2	9	8.8	11.7	93	6
	All	23	94	44.5	65.0	170	122
Highest prevalence	Mild	9	29	23.5	25.2	138	96
	Mod	11	25	15.4	11.8	95	47
	Severe	2	23	22.4	30.5	95	65
	All	22	26	19.3	19.2	113	72
All states in USA	Mild	33	115	49.9	60.5	203	130
	Mod	44	82	27.0	24.5	137	104
	Severe	9	71	10.5	14.5	96	55
	All	86	94	34.1	43.4	158	116

Within the table, each region is sub-divided into *Severity* groups. The severity ratings are simply *Mild, Moderate and Severe* along with a row for *All* severity groups within a region combined. *Severity* was a subjective measure noted by each mother in the survey. It is not an official medical opinion although it was certainly influenced by the professionals consulted by mothers.

The results table shows *n* as the number of participants in each subgroup. The *Tap Water* column represents the average sulfate concentration of tap or well water reported in mg/L. *Water Mix* is the average sulfate concentration of the water mixture actually consumed by the mothers. It is a weighted average based on the number of glasses per day estimated for each type of water. It differs from the tap value because drinking water is commonly filtered and/or supplemented by bottled water. The standard deviation is listed next. The final columns switch to milligrams as units for the total sulfate reported from beverages plus water along with the standard deviation. *Bev & Water* was calculated by adding the sulfate concentration times the amount that was drunk for each beverage and type of water. All sulfate data points are means of *n* samples.

To better understand the results table, it helps to note a few facts about water and beverages. The 2003 EPA report estimates the median public water system across the country to have a sulfate concentration of 24 mg/L. The range is quite wide from zero to above 600 mg/L indicating that the mean would be higher than the median, although no estimates are given. The sulfate obtained from 2 L of median public water would be 48 mg. Beverages like coffee, tea, milk and juice are discussed by a US Department of Agriculture data brief titled *Beverage Choices of U.S. Adults.* Using this information (but reducing soda and alcohol for pregnant mothers) provides an estimate of 90 mg of sulfate per day from beverages (LaComb et al. 2011). Combining, 138 mg would be an estimate for sulfate from beverages plus water for a typical pregnant woman.

### Results in Graphical Form

**Fig. 1:**
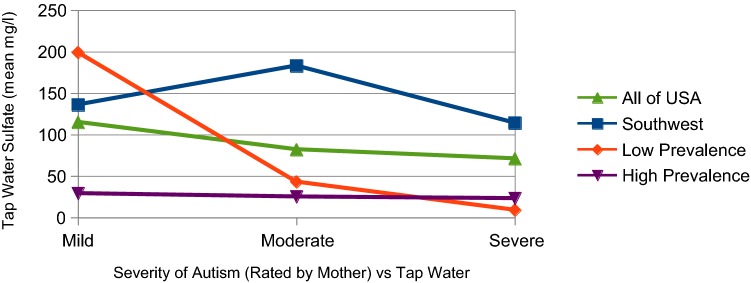
Tap water sulfate levels in selected regions

The results table is best visualized with the help of line graphs. Figure 1 plots *Tap Water s*ulfate concentrations for each of the four study regions as a function of autism symptom severity. The data lines are widely separated but unsurprising. The Southwest shows relatively high sulfate simply because it is served by Colorado River water which is naturally high in minerals. The High Prevalence region reports the lowest sulfate values in this study, reaffirming water quality reports from major cities for these states. The Low Prevalence region indicates high sulfate for mild conditions, dropping sharply for more severe autism. While the USA as a whole shows a flat, mid-range plot with little apparent association with symptom severity.

**Fig. 2:**
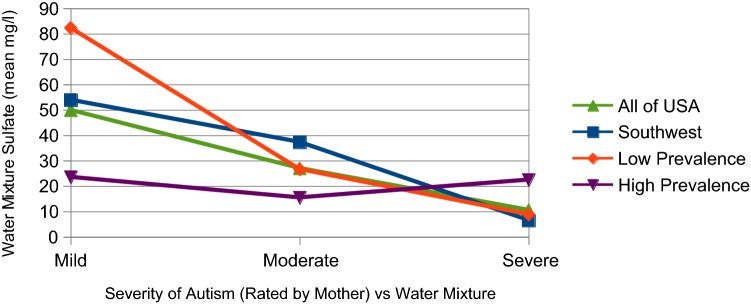
Water mixture reported by mothers

Figure 2 plots the sulfate concentrations for the *Water Mix* actually consumed by the pregnant mothers. This includes tap or well water, filtered and unfiltered, along with a variety of bottled waters. Again, each of the four regions is plotted against autism severity. These curves are surprisingly different from tap water alone. Three of the four regions suggest a trend, hinting that mild symptoms and higher sulfate may be related. High Prevalence is the outlier, more or less flat but on the low end of sulfate. Standard deviations for the data points are shown in Table 3.

**Fig. 3:**
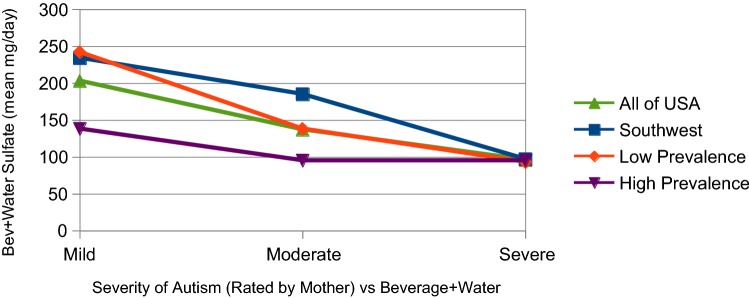
Beverages plus water reported by mothers

The sum of sulfate from *Bev & Water* is presented in Fig. 3. These are the beverages, tap water and bottled waters that were drunk by the participating mothers. In this case, sulfate is not a concentration but the mg weight of the sulfate consumed daily. It was calculated from the number of 8 oz glasses reported by each mother for various types of beverages and water. As in previous figures, each of the regions is plotted against autism severity. All of the regions now show a monotonic, decreasing function clearly associating severity with lower levels of sulfate. These relationships are examined in the regression analysis that follows.

### Regression, Correlation and Comparison

The appearance of the plotted data suggests a reasonable correlation between sulfate consumed by pregnant mothers and the resulting severity of autism shown in their children. Of course, this assumption needs to be tested for statistical significance. Table 4 presents the relevant data for the Beverage plus Water graph. Each of the four regions is characterized by *n*, the number of data sets, and by the *Mean ± SD* (standard deviation) of sulfate for all levels of autism severity. Then a linear regression is performed on the data sets to minimize the sum of the squared errors. To perform the numerical calculations, autism severity is mapped in the following manner: mild = 1, moderate = 2 and severe = 3. The resulting Pearson Linear Correlation Coefficient is noted as *r* in the table along with a description of the *Strength of Correlation*. In the *Strength* column, *p* is the probability that the correlation is a result of statistical chance and therefore invalid. For small sample sizes in our study, *p* is not insignificant but drops to < 1% for the full USA.

**Table 4:** Correlations between sulfate and severity for beverage plus water

Beverage plus water linear correlation (Sulfate plotted against severity of autism)				
Region	n	Mean ± SD (mg sulfate)	r (Pearson)	Strength of correlation
Southwest region	32	193 ± 133	− 0.35	Low inverse p < 0.05
Lowest prevalence	23	170 ± 122	− 0.44	Low-moderate inverse p < 0.05
Highest prevalence	22	113 ± 72	− 0.27	Low inverse p < 0.25
All regions of USA	86	158 ± 116	− 0.32	Low inverse p < 0.01

The correlations are negative indicating sulfate is inversely related to autism severity, lower sulfate resulting in higher severity. However, the relationship is not strong, as the correlations are typically rated as low. Such results might be expected noting the wide spread of data. But even a low signal seems significant given the unknowns in the study. The genetics of the mothers and children were completely unknown, other than the generalization that autism resulted. Sulfate from food sources was not tracked, only sulfate in drinking water and beverages. Since the typical age of the child in the study was 6 years, memory errors may have occurred. And the water tested was therefore many years out of date. Finally, the mothers reported typical water and beverage consumption that may have been representative, but this may have varied over the course of a full pregnancy. With all of this uncertainty, the actual survey results are surprisingly clear. It is worth noting that the study was conducted in four dissimilar regions with widely differing levels of sulfate in tap water and very different rates of autism. In essence, this was four studies rolled into one. And all four showed a similar correlation between sulfate and autism.

**Fig. 4:**
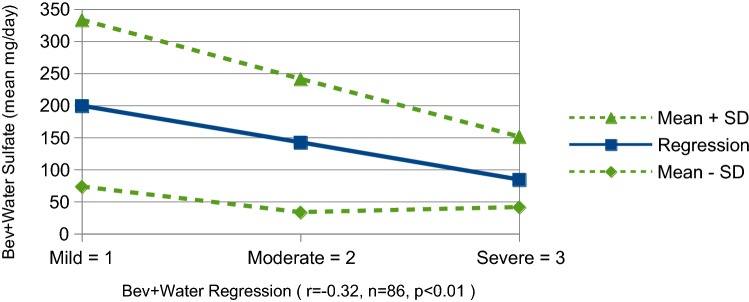
Linear regression of beverage plus water for USA

A graph is shown in Fig. 4 to better visualize the relationship, data spread, regression and correlation. It is the plot of sulfate in Beverage plus Water vs Autism Severity for data covering the full United States. Severity mapping to the sequence 1, 2, 3 is depicted along with single standard deviation error bands and the resulting linear regression line. For this set of 86 samples, the correlation is r = − 0.32 with the probability of a statistical flaw being p < 0.01, < 1%. In a single figure, this is a snapshot of the results of our study.

Welch unequal variance t-tests can be applied to tap water, testing the statistical validity of sulfate differences between regions. To focus on water alone, we include all reported values of sulfate in tap water without regard to autism severity. The Southwest region was selected for its link to Colorado River water and probable high mineralization. Our survey reports a Southwest sulfate mean of 151 mg/L compared to 59 mg/L for the other regions combined. The probability of this difference as untrue is p = 0.00037, confirming our assumption of higher than average sulfate levels for the Southwest. The Southwest will be examined further in the discussion section.

The eight states with the lowest rates of autism form the Low Prevalence region which reports a tap water sulfate mean of 94 mg/L. The eight states with the highest rates of autism form the High Prevalence region with a sulfate mean of 26 mg/L, just 28% of the Low Prevalence average. Applying a Welch *t* test, the null probability of this difference as untrue is p = 0.059. Though on the cusp of the usual 5% benchmark, this is an indicator of the importance of sulfate considering tap water values only represent the water that is available, not the water mix actually consumed. It is significant that the prevalence regions were selected using Department of Education autism data alone. Yet they show a definite association with local tap water sulfate concentrations.

## Discussion

How could a deficit of a few hundred milligrams of sulfate make any difference in the diet of a pregnant woman? There are some clues in the medical literature. In Waring’s English study 19 years ago, urine was analyzed for those with autism and compared against neurotypicals. The results showed autistic urine to contain 6800 uM sulfate compared to a normal reading of 3000. Assuming daily urine discharge at 1.4 L, the extra sulfate in urine for those with autism was 510 mg per day. This suggests that tissue in those with autism may be starved for sulfate. And this assumption is confirmed by Adam et al.’s (2011) Arizona study showing blood levels are below normal, only 35% in the case of free sulfate. Note that sulfate in drinking water is typically in the form of a free ion, exactly what seems to be missing in those with autism.

Low blood sulfate coupled with high levels in urine is likely due to poor reabsorption in the kidney. Waring notes that the proximal tubule of the kidney contains transporter proteins necessary for sulfate reabsorption. Loss of these transporters leads to renal sulfate wasting and reduced blood sulfate level. In particular, two proteins deserve mention, NaS1 (sodium-sulfate co-transporter SLC13A1) and SAT1 (anion exchanger SLC26A1). Located in the kidney, they move sulfate from urine at the apical membrane, then back into the bloodstream at the basolateral membrane. It is interesting to note that NaS1 expression is regulated by vitamin D. In a study of VDR knockout mice with diminished vitamin D levels, urinary sulfate excretion increased by 42% and blood serum sulfate decreased by 50% (Bolt et al. 2004). Since vitamin D deficiency is quite common, affecting 41.6% of the US population as shown in the National Health and Nutrition Examination Survey of 2005/2006, it may be an environmental factor for dysfunctional sulfate levels (Forrest and Stuhldreher 2011). Taking a clue from vitamin D dependence on sunlight exposure of the skin, MIT researchers have noted a link between autism and sunlight available during the third trimester of a pregnancy. Pregnancies in northern latitudes have a greater risk of autism if the birth is timed to late winter or early spring when low levels of available sunlight prevail (Hartzell and Seneff 2012).

Is a sulfate deficit in water a significant environmental trigger for autism? That is the question underlying this study. The Southwest region was chosen for its potential to disprove sulfate’s importance. Both Southern California and Central Arizona are served by Colorado River water, high in sulfated minerals, yet they are in the mid-range for autism prevalence. Looking at tap water, participants in the Southwest region reported an average concentration of 151 mg/L sulfate. As expected, that is quite high. But the water mixture consumed by the mothers measured just 40 mg/L, only 26% of that available from tap. This offers support for sulfate’s importance. In retrospect, it might be assumed that water with a high mineral content would lack the fresh taste of spring water and this might result in widespread replacement with purified water.

Bottled water is an interesting modern phenomenon, rare 70 years ago when autism was virtually unknown but very common in today’s world. If participants in the Southwest region had rejected bottled water and had drunk 2 L of local tap water instead, 222 mg of sulfate would have been added to their diet. Bottled water is not the only factor that has reduced sulfate in the modern world. Since the enactment of the Clean Water Act of 1972, the EPA has been tasked with cleaning up public water in the United States. Clearly, this is good for the country as it minimizes the microbes and toxins that pose health hazards. Of course, water that has been cleaned to contain fewer contaminates will naturally contain less sulfate. The reduced sulfate content of some tap water and most purified bottled water may be relevant when considering the potential role of a low sulfate supply in autism.

Many simple steps may be taken to reverse this trend and increase sulfate in the diet of pregnant women. As discussed above, if local tap water is mineral rich, it could be used instead of purified, bottled water. If taste is an issue, the use of simple carbon filters like Brita effectively improves the flavor while leaving sulfate largely intact. If local tap water is low in sulfate, bottled mineral water may be substituted. An interesting option is Pellegrino because it provides 408 mg of sulfate in each liter bottle. Currently, it is owned by Nestle Foods and widely available across the globe. Many other options for mineral water are listed in Table 2.

Sulfate supplements are inexpensively available. Most common are the heptahydrate versions of ferrous and zinc sulfate. Ferrous sulfate is often sold at a strength of 65 mg iron, providing 112 mg of sulfate. Zinc sulfate is usually sold at a strength of 50 mg zinc, resulting in 74 mg sulfate. However, they provide about three times the daily value of iron or zinc which may limit their usefulness. Another option is Epsom salts (magnesium sulfate) used for both drinking and bathing. One quarter level teaspoon (1.33 g) of the common heptahydrate version yields 518 mg sulfate and 131 mg magnesium. When dissolved in 2 L of purified water, a mineralized water is created with a sulfate concentration similar to that of the Colorado River. To circumvent digestive issues, Epsom salts may be added to bath water. Transdermal absorption has been anecdotally reported to increase body sulfate levels (Adams et al. 2018). Of course, all supplements taken during pregnancy should be approved by a physician.

Some food contains significant amounts of sulfate and may offer a natural choice for increasing sulfate in the diet. Table 5 lists the foods and beverages with the highest levels of dietary sulfate upon digestion. The data are from Florin’s study which preps samples using acid hydrolysis to simulate gastric acid. The table entry “Reference at 24 mg/L” refers to the EPA published median for sulfate in public water. Using a combination of tap water, bottled mineral water and select foods or beverages, it’s not difficult or inconvenient to make up for the additional 510 mg of sulfate measured in the urine of individuals with autism.

**Table 5:** Foods and beverages high in dietary sulfate

Foods and beverages high in dietary sulfate (Sulfate means per Florin with typically four or more samples)			
Food name	Serving	Grams (g)	Sulfate (mg)
Broccoli	1 cup	90	81
Cabbage	1 cup	90	72
Whole wheat bread	1 slice	36	54
Raisins	¼ cup	40	52
Dates	6 dates	40	44
Avocado, fresh	½ avocado	80	42
Potato, baked	1 potato	120	37
White bread	1 slice	28	36
Yogurt	¾ cup	160	30

Beverage	Serving (oz)	Liters (l)	Sulfate (mg)
Tomato juice	6	0.18	45
Grape juice	6	0.18	36
Cola soft drink	12	0.36	29
Milk, cow or soy	8	0.24	24
Coffee, less water	8	0.24	24
Tea, less water	6	0.18	18
Non-cola soft drink	12	0.36	14
Apple or citrus juice	6	0.18	13
Reference at 24 mg/L	8	0.24	6

The drinking water study described by this paper targets the water and beverages consumed during pregnancy, a crucial time for brain development. Of course, the diet of young children must be important, too. That is why the sulfate concentration of the water mixture used by the mother is tracked. This is the water most likely available to other members of the family, including infants and developing children. Overall, this study suggests that a low sulfate supply in drinking water and beverages consumed during early development may be associated with an increased risk of autism and its severity.

## Conclusion

Our survey of the drinking water and beverages of mothers of children with autism showed an association between sulfate deficit during pregnancy and autism severity. There was a clear dose versus response relationship suggesting that sulfate during pregnancy may be helpful in reducing the severity of autism. Since the same water was available to the entire family, sulfate during infancy also may be helpful in reducing the severity of the condition. Comparing the eight states with the highest rate of autism to the lowest, prevalence is reduced by a factor of almost three according to Department of Education statistics. Since there is a large difference in the available sulfate in water from these regions, it may be possible to significantly reduce the incidence of autism by supplementing with sulfate rich food and water during pregnancy and early childhood. Based on losses in urine, 510 mg of sulfate per day may be an appropriate goal for pregnant women with an elevated risk of autism.

## Electronic supplementary material

Below is the link to the electronic supplementary material.

Supplementary material 1 (XLS 324 kb)
